# Potential Drug-Related Problems in Pediatric Patients—Describing the Use of a Clinical Decision Support System at Pharmacies in Sweden

**DOI:** 10.3390/pharmacy11010035

**Published:** 2023-02-14

**Authors:** Sazan Abass Abdulkadir, Björn Wettermark, Tora Hammar

**Affiliations:** 1 Department of Pharmacy, Faculty of Pharmacy, Uppsala University, 752 37 Uppsala, Sweden; 2 The eHealth Institute, Department of Medicine and Optometry, Linnaeus University, 391 82 Kalmar, Sweden

**Keywords:** clinical decision support system, EES, dispensing, pharmacy, alerts, drug-related problems, pediatric

## Abstract

The clinical support system Electronic Expert Support (EES) is available at all pharmacies in Sweden to examine electronic prescriptions when dispensing to prevent drug-related problems (DRPs). DRPs are common, and result in patient suffering and substantial costs for society. The aim of this research was to study the use of EES for the pediatric population (ages 0–12 years), by describing what types of alerts are generated for potential DRPs, how they are handled, and how the use of EES has changed over time. Data on the number and categories of EES analyses, alerts, and resolved alerts were provided by the Swedish eHealth Agency. The study shows that the use of EES has increased. The most common type of alert for a potential DRP among pediatric patients was regarding high doses in children (30.3% of all alerts generated). The most common type of alert for a potential DRP that was resolved among pediatrics was therapy duplication (4.6% of the alerts were resolved). The most common reason for closing an alert was dialogue with patient for verification of the treatment (66.3% of all closed alerts). Knowledge of which type of alerts are the most common may contribute to increased prescriber awareness of important potential DRPs.

## 1. Introduction

Drug-related problems (DRPs), such as adverse drug events (ADEs), are common [1]. DRPs result in patient suffering and substantial costs for society [2]. They can even lead to death [2]. DRPs are frequent and are either circumstances or events that interfere, or have the potential to do so, with the accomplishment of the optimal outcome of a drug treatment [3]. In contrast, medication errors are failures in any of the steps in the medication management process, i.e., prescribing, dispensing, or administering a drug [4]. Medication errors may cause patient harm, while adverse drug events (ADEs) relate to actual harm from using a drug [4]. Medication errors and potential ADEs may relate to inadequate information regarding the prescribed drug and/or the patient, indicating the importance of needed information being available [4].

Pharmacists working in community pharmacies are often the last health care provider in the chain of care, which makes them valuable for preventing DRPs as well as detecting prescription errors [5]. Modifications performed by pharmacists of prescription errors has been shown to be of clinical value, as well as clinical pharmacists’ interventions [4,6]. DRPs and dispensing errors can be further avoided by using a clinical decision support system (CDSS) in connection with the dispensing [7]. In Sweden, most parts of the medication management process are handled digitally [2]. Prescriptions have for a long time been transferred and stored electronically [3,8]. The Swedish eHealth Agency store electronically transferred prescriptions in a prescription repository. This system paved the way for the utilization of IT support, i.e., CDSS, when dispensing a prescription [9].

A CDSS provides knowledge combined with patient specific information presented at appropriate times using knowledge-based rules and algorithms [10]. The use of a CDSS in medication management processes enables linking of patient-specific factors with current prescriptions, thus supporting health care professionals in detecting potential DRPs. Although CDSS has the potential to improve medication safety, the results of using a CDSS vary [6,11,12], and may be affected by the design of the system, implementation, and clinical relevance of generated alerts, among other things [6]. Decisions on whether to implement CDSS in health care should be based on considerations of whether the desired safety effects will provide more benefit than the risk of alert overrides. Alert overrides means that the health care provider may not follow information presented due to alert fatigue, caused by alerts that are not clinically relevant or an unreasonable number of alerts [13]. Thus, clinically relevant alerts are at a risk of being missed [13]. Other risks are disturbance of the workflow and diminishing medical judgement [6]. Most CDSS described in the literature are aimed at prescribers in health care, but there is also some research regarding CDSS for community pharmacists [14,15].

The CDSS called the Electronic Expert Support (EES) is a government-owned system [7] available at all pharmacies in Sweden with the aim to increase patient safety as well as improve drug use [9]. It enables identification of drug–drug interactions (DDIs) and provides warnings when dosages deviate from the correct dosage among other things [9]. Incoming prescriptions are analyzed by the system before being dispensed, resulting in quality assurance. The EES system is developed and maintained by the Swedish eHealth Agency with the goal to enable safer prescribing and to pave the way for patient-specific counseling [9]. The knowledge database in the EES system is constantly updated [2,16]. The clinical relevance of alerts provided by the EES was explored in a study carried out in 2015, in which physicians reported that they perceived 68% (502/740) of the alerts as clinically relevant [10]. A study from 2020, which examined the importance of using EES in a Swedish community pharmacy, indicated that the system contributes to the identification and resolution of DRPs [3]. Some weaknesses with EES that have been described include limitations in the relevance of alerts, a lack of patient-specific information (i.e., diagnoses and laboratory values from the electronic health records), and errors in the medication list utilized for EES analysis (i.e., non-current prescriptions or missing medications) [7,10]. The EES alerts for high dose for pediatric patients are, however, based on rules that include medication with recommended doses based on the child’s age and in other cases weight, and oftentimes structured in age ranges [9].

Medication amongst children is challenging [17,18,19]. They cannot be treated as small adults due to important pharmacokinetic differences, e.g., the surface area for drug absorption and pH [20]. The developmental phases and growth that take place during childhood make it of great importance to ensure the adequate treatment of all children [20]. Off-label drug use, when drugs are prescribed outside their licensed indications [21,22], is common in pediatric drug treatment [17,22]. The underlying causes of off-label drug use are limitations in the pediatric drug development and insufficient evidence-based treatment recommendations for many drugs [17,22], resulting in formulations that are used by children not adapted to them [22,23]. Thus, there is uncertainty about the effects and side effects of a treatment. Around 30–40% of this patient group have had at least one DRP [17]. The occurrence of DRPs in pediatric patients is thus a major concern and is mostly related to prescribing, i.e., drug selection, usage, and dosage [17]. It is, however, challenging to determine any harmful consequence of DRPs, since the medications that are administered to a child are mostly tracked by the parents and/or caregivers [20]. Community pharmacists have an important role in preventing DRPs in pediatric patients [24], but studies indicate that more training or better support is needed for pharmacists to be able to detect and prevent errors in this population [25,26]. Furthermore, there is limited knowledge about CDSS to prevent DRPs in pediatric patients in the community pharmacy setting [27,28].

A pharmacist performing an analysis using EES will receive an alert in cases where the system detects a potential problem [3]. The types of alerts generated for pediatric patients at Swedish community pharmacies when using the EES system has not been studied before to the best of our knowledge. This study is intended to provide new knowledge in this area, as well as providing an overview of the use of EES over time.

The aim of this study was to examine the use of the decision support system EES at pharmacies in Sweden for the pediatric population (ages 0–12 years), by describing what types of alerts for potential DRPs are generated, how they are handled, and how the use of EES has changed over time. More specifically, we wanted to study

- The number and proportion of children (ages 0–12 years) receiving EES analyses over time;
- The type of alerts for potential DRPs being generated by EES for pediatric patients;
- The proportion of EES alerts being resolved for pediatric patients and examine potential differences between the types of alerts;
- What kinds of actions were taken to resolve the generated alerts for pediatric patients.

## 2. Materials and Methods

The study used quantitative data from the Swedish eHealth Agency delivered on an aggregated level and analyzed to answer the research questions in the current study. The data are automatically generated and compiled into Excel files at the Swedish eHealth Agency on a weekly basis.

### 2.1. Timeline

Three different types of data were obtained from week 11 of year 2020 to week 11 of year 2022 for the total population and the pediatric population. The available data determined what types of measurements could be taken, resulting in different results being presented in this study for the total population compared to the pediatric population. Two different types of data were obtained from week 11 of year 2022 for the pediatric population. For details see Table 1. The design of the study was partly based on a Swedish study from 2020 describing the same decision support system but for another population and timeline [7].

### 2.2. Setting

This study included all pharmacies in Sweden, approximately 1400 pharmacies in total. All individuals that were dispensed a prescription at a pharmacy were included. In this study the term pharmacist refers to the two following categories of licensed pharmacy practitioners in Sweden: a prescriptionist with three years of university education and a pharmacist with five years of university education. Both have similar legal rights and obligations.

### 2.3. Electronic Expert Support System (EES)

EES is a rule-based decision support system (a so-called expert system), which reflects the clinical parameters/conditions for a particular drug [3,9]. It was developed by Medco Health Solutions, USA, and later adapted to Swedish clinical practice [2,3]. The system consists of approximately 9000 rules [9]. Some rules include every drug within a therapeutic class, whilst other rules only cover one drug form of a substance [9]. The rules are divided into the nine following main categories of alerts: high dose, drug gender warning, therapy duplication, drug–drug interactions, drug–disease inferred, elderly patients, pediatric patients, fetal effects, breastfeeding [7].

The categories elderly and pediatric have two additional warnings being, respectively, “high dose elderly” and “age warning elderly” and “high dose pediatric” and “age warning pediatric” [9]. The EES alerts are displayed in different ways depending on the integration and interface of each prescription dispensing system. The information is thus displayed solely as the main categories, elderly or pediatric, without further dividing the information into “high dose” and/or “age warning” [9].

When the EES system is used, an analysis is made for each prescription from a national medication list based on the rules in EES [3,7]. The prescriptions included in an EES analysis are current prescriptions with medication left for dispensing as well as prescriptions that are no longer valid if they have recently been dispensed if EES estimates the patient still has drugs at home [7].

To perform an EES analysis an active choice is required [7]. In previously conducted studies, patient consent was additionally required in order to use EES. It has repeatedly been mentioned as problematic due to being an obstacle when performing an analysis [7]. The need for patients’ consent was, however, removed on June 2nd, 2020, after being reassessed from a legal point of view [29]. Since the availability of the EES system in 2010, the initial low level of use [5] has continuously increased [7]. A Swedish study in 2020 observed a long-term increase in the usage while studying the effects of a national intervention [7]. It is however not certain that the observed increase would not have taken place without the intervention [7].

### 2.4. Data and Statistics on the Use of EES

The use of EES, i.e., the number of EES analyses, is calculated as follows: A customer is counted only once per day and per pharmacy. Thus, performing an EES analysis in the morning and another one in the afternoon with the same customer and at the same pharmacy is calculated only once. If the customer goes to different pharmacies in the morning and in the afternoon where an EES analysis is performed each time it will be calculated as two EES analyses, one at each pharmacy. The statistics in EES analyses are based solely on the use of EES, i.e., it does not matter if the prescription was dispensed or not. The number of resolved alerts, i.e., alerts being closed, describe the alerts that are in the system on Monday morning at 6:00 am. This requires that the alerts are still “active”. At least one prescription needs to be current for an alert to remain in the system.

The data on the closed alerts have been assigned specific acronyms. The explanation of the alert categories is as follows: DD1 (D interaction—clinically relevant interaction. The combination is best avoided.), DD2 (C interaction—clinically relevant interaction that can be handled, e.g., by dose adjustment), DD3 (A and B interaction—minor interaction of no clinical relevance and clinical outcome of the interaction is uncertain and/or may vary), DXI (drug–disease inferred—potential contraindications for a drug due to existing inferred diseases) GSX (drug gender warning), HD2 (high dose—a prescribed dose exceeding the maximum daily dose), HDP (high dose pediatric—a prescribed dose exceeding the maximum daily dose for pediatric), PAP (age warning pediatric), and TD1 (therapy duplication—dispensing of two or more drugs within the same therapeutic class), and TR1 (not a specific category). TR1 is not a specific category but is called “Supplementary review” and has been created to distinguish certain alerts from their respective categories.

For parts of the analysis, five reference weeks were chosen during the period for which data were collected: it was week 11 of year 2020, week 36 of year 2020, week 11 of year 2021, week 36 of year 2021, week 11 of year 2022. The reference weeks were chosen to be as representative as possible, i.e., no holidays or events. Data on the use of EES from previous studies show for example a decrease in the use during June to August [7].

#### 2.4.1. EES: Closing an Alert

Pharmacists handle and make decisions based on the generated alerts and have the option to close alerts after being handled [9]. Closing an alert will result in the alert not being generated next time an analysis with EES is performed on the same medications. Closing an alert requires the pharmacist to document a choice of the action taken. There are six available choices of action in the system to choose from: dialogue with patient—verification of treatment; dialogue with patient—cancellation of prescription; dialogue with patient—referral to prescriber; contact with prescriber—without changing prescription; pharmaceutical assessment only; other action [9]. 

#### 2.4.2. National Intervention

National interventions called “focus weeks” are initiated by the Swedish Pharmacy Association, and have been conducted each year starting in 2018 with a chosen focus each time [7,30]. The nationwide interventions were directed at increasing the use of the EES and knowledge of the EES system but have focused on different areas:

- Year 2018 (week 15), focused on the elderly (aged 75 years or older);
- Year 2019 (week 15) focused on the elderly (aged 75 years or older);
- Year 2020 (week 43) focused on the pediatric population (aged 0–12 years);
- Year 2021 (week 16) focused on therapy duplication;
- Year 2022 (week 14) focused on DDIs.

### 2.5. Data Analysis

Data were analyzed using Excel. In total, the data included 106 weeks. Major events that may have affected the use of EES, i.e., the number of EES analyses, were highlighted (can be seen as vertical dotted lines in some of the figures). These major events were when the new law on a national medication list came into force, the need for patients’ consent to perform an EES analysis was removed, and when the focus weeks took place (one focus week each year).

The statistics on the number of prescriptions dispensed and the number of EES analyses is displayed for the total population and for the pediatric population. The pediatric population cover however slightly different age groups. The number of prescriptions dispensed cover ages below 13 years, i.e., all children who have not reached the age of 13 are included. The number of EES analyses cover ages 0–12 years, i.e., until the day the child turns twelve. The calculation was made on the date of birth, resulting in a difference of approximately one year.

### 2.6. Ethics Statement

The data obtained from the Swedish eHealth Agency were extracted, delivered, and handled in an aggregated form with no data on the individual level. The study did not include any intervention or changes to the ordinary work at pharmacies. The researchers did not affect the use of the EES system, and only analyzed data generated. Thus, ethical approval was not necessary.

## 3. Results

The use of EES, i.e., the number of EES analyses, has increased markedly during the period March 2020–March 2022, both for the total population and the pediatric population. 

### 3.1. The Use of EES

**Figure 1:**
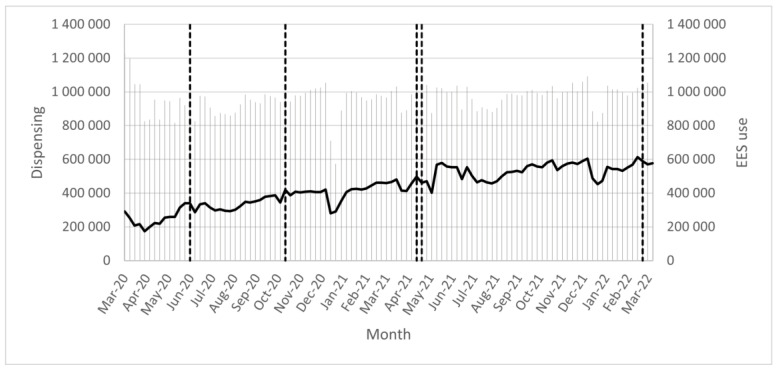
For total population—number of dispensed prescriptions (grey clustered columns) in relation to the number of Electronic Expert Support (EES) analyses (black line) per week between week 11, 2020 and week 53, 2020 (month = Mar-20–Dec-2020); week 1, 2021 and week 52, 2021 (month = Jan-21–Dec-21); week 1, 2022 and week 11, 2022 (month = Jan-22–Mar-22). Events that may have affected the use of EES that have taken place are indicated as dotted lines.

**Figure 2:**
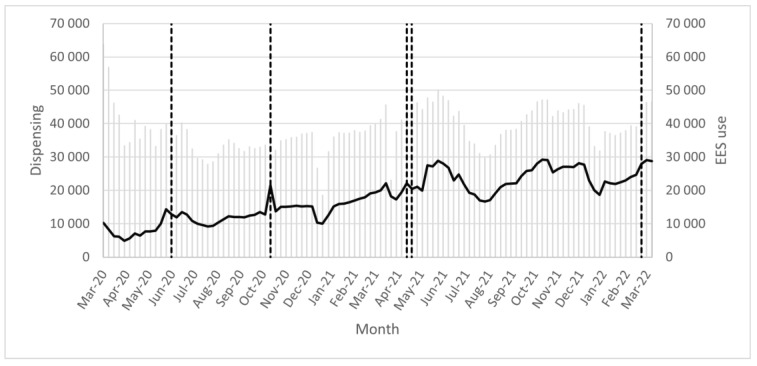
For the pediatric population (ages 0–12 years)—number of dispensed prescriptions (grey clustered columns) of the pediatric population in relation to the number of Electronic Expert Support (EES) analyses (black line) per week between week 11, 2020 and week 53, 2020 (month = Mar-20–Dec-2020); week 1, 2021 and week 52, 2021 (month = Jan-21–Dec-21); week 1, 2022 and week 11, 2022 (month = Jan-22–Mar-22). Events that may have affected the use of EES that have taken place are indicated as dotted lines.

Events that may have affected the use of EES, i.e., the number of EES analyses, were marked in figures following weeks (Figure 1 and Figure 2). The need for patients’ consent to perform an EES analysis was removed week 23, 2020 (month = June-20), indicated as the first dotted line from the left. The focus week in 2020 was week 43 (month = Oct-2020), indicated as the second dotted line from the left. The focus week in 2021 was week 16 (month = Apr-21), indicated as the third dotted line from the left. The new law on a national medication list came into force week 17, 2021 (month = May-21), indicated as the fourth dotted line from the left. The focus week in 2022 was week 14 (month = Apr-22), indicated as the fifth dotted line from the left. The five reference weeks used in this study was week 11 of 2020 (month = Mar-20), week 36 of 2020 (month = Sep-20), week 11 of 2021 (month = Mar-21), week 36 of 2021 (month = Sep-21), and week 11 of 2022 (month = Mar-2022). Summer holidays in Sweden occurred during June to August and Christmas holidays occurred at the end of December.

There are large variations in the use of EES during the period analyzed (Figure 1 and Figure 2). The level of EES use decreased markedly during holidays, especially during summer and Christmas. The number of analyses increases with each focus week; however, the use does not continue at the same level, as seen by the drop in the number of analyses right after the focus weeks.

The focus week during week 43 of year 2020 focused on pediatrics. This coincides with the clear increased EES use in children during this week. The number of analyses per week was 12,802 in week 42 of 2020 and 21,527 in week 43 of 2020, resulting in an almost twofold increase (Figure 2). See Supplementary Material (Figure S1) for a comparison between the numbers of EES analyses of the total population and the pediatric population.

The amount of dispensing maintains a relatively even level, in comparison with the increased level of EES use as seen by the change of the black line (Figure 1 and Figure 2). The amount of dispensing and the level of EES use decreased during holidays, especially during summer and Christmas.

### 3.2. EES Analyses and the Numbers of Alerts Generated and Resolved

When comparing the number of EES analyses and the proportion of individuals receiving an EES analysis during each of the five reference weeks there is a clear increase between each week for both the total population and the pediatric population (Table 2). For patients of all ages, the number of EES analyses in week 11 of year 2022 was 576,510, which represents almost 57% of those having prescriptions dispensed that week (Table 2). For patients aged 0–12 years, the number of EES analyses week 11 of year 2022 was 28,748, which represents almost 57% of those having prescriptions dispensed that week. 

The same week, alerts that were closed in the system of patients aged 0–12 years represented almost 2.8% of the alerts. Each time the pharmacists use EES, one or more alerts can be generated. The number of EES alerts for patients aged 0–12 years was 30,870 during week 11 of year 2022, which gives an average number of alerts per EES analysis of 1.1. The numbers and proportions of EES analyses varied during the five weeks of measurements, but similarity in the changes of numbers of patients receiving an EES analysis are observed when comparing patients of all ages and patients aged 0–12 years. See Supplementary Materials (Figure S2) for a comparison between the numbers of alerts resolved, i.e., alerts being closed, of the total population and the pediatric population.

### 3.3. EES Analyses and the Numbers of Alerts Generated and Resolved for Pediatrics

**Figure 3:**
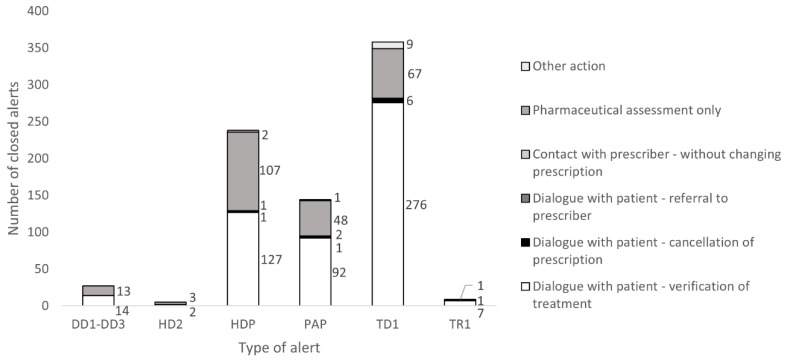
Number of closed alerts per category for the pediatric population aged 0–12 years during week 11 in March 2022 illustrated with the action taken by the pharmacists. Explanation of alert categories: DD1 (D interaction), DD2 (C interaction), DD3 (A and B interaction), HD2 (high dose), HDP (high dose pediatric), PAP (age warning pediatric), TD1 (therapy duplication), and TR1 (not a specific category).

The most common type of alert for a potential DRP that was resolved, i.e., alerts being closed, among pediatric patients aged 0–12 years during week 11 of 2022 was therapy duplication, for which 4.6% of alerts were resolved (Table 3). The most common type of action to resolve this type of alert, i.e., reason for closing this alert, was dialogue with patient for verification of the treatment, representing 77% of documented actions (Figure 3). High dose pediatric and age warning pediatric are alert categories with many closed signals. The two most common reasons for closing an alert are dialogue with patient for verification of the treatment and pharmaceutical assessment (66.3%; 30.6%).

The most common type of generated alert for a potential DRP among pediatric patients aged 0–12 years during week 11 of 2022 was high dose pediatric (30.3% of all alerts) (Table 3). Other categories with large numbers of generated alerts are therapy duplication, age warning pediatric, and drug–drug interaction (25.2%; 23.8%; 18.2%).

## 4. Discussion

To our knowledge, this nationwide study is the first analyzing the type of alerts generated by the EES system for the pediatric population with a focus on how these were resolved, i.e., the reason for closing the alert. This study shows that the number of EES analyses increased for both the total population and the pediatric population (ages 0–12 years). The number of closed alerts increased as well, although the proportion of alerts being closed was still as low as 2.5% of all alerts in 2022. The most common type of generated alert for a potential DRP among pediatric patients was high dose pediatric (30.3% of all alerts generated). The most common type of alert for a potential DRP that was resolved among pediatrics was therapy duplication (4.6% of alerts being resolved). The most common reason for closing an alert was dialogue with patient for verification of the treatment (66.3% of all closed alerts).

### 4.1. The Increasing Use of EES and Possible Explanations

Despite the large variations in the use of EES during the period analyzed, the statistics showed an increase during each focus week. Results from a previous study in 2020 [6] showed a similar rapid increase in the use of EES when analyzing any effects of the focus week in year 2018 (week 15). Data in the same study showed similar decrease during summer holidays (June to August) and Christmas (the end of December). Despite the aim of increasing the use of EES by implementing the focus weeks, the increase in the use is not constant. The use of EES drops right after each intervention, but observing the use during that year after an intervention compared with before shows a long-term effect. A similar observation has been seen in the study in 2020 [7].

The proportion of the pediatric population receiving an EES analysis in week 11 of year 2022 seems to be quite similar to the proportion for the total population (Table 2). Comparing the proportion of individuals of all ages receiving an EES analysis to a study carried out on the use of EES [7], an increase can be observed from 19% (week 11, 2019) to 57% (week 11, 2022).

It is not feasible to analyze any effect of the removal of the need for patients’ consent to perform an EES analysis due to the timeline of the obtained data. Data begin from week 11 of year 2020 and the consent was removed week 23 of year 2020. However, in previous studies [7], this was repeatedly mentioned as problematic. Thus, the removal of the required consent may have contributed to the major increase in the use of EES since then. 

On 1 May 2021, a new law on a national medication list (NLL) came into force in Sweden, providing a nationwide source of information about a patient’s prescribed and dispensed medications [30]. The proportion of individuals receiving an EES analysis of the total population during the reference week prior to the new law on a national medication list (reference week 11, 2021) was 46%. Comparing to the following reference week (week 36, 2021), the proportion was higher, about 54%. However, with the results from the current study it is not possible to conclude any effects of the new law. 

### 4.2. Challenges with Non-Relevant Alerts Generated

One of the main challenges with implementing a CDSS is the risk of non-relevant alerts taking up time and attention, and EES is not an exception. One reason for some alerts not being relevant is that analyses are based on a list of prescriptions that contains many errors and non-current medications. One recent study showed that one third of patients had at least one discrepancy in their list of prescriptions, which is the same list used to generate EES alerts [31]. A shared medication list can improve the relevancy of CDSS alerts [7]. If prescribers and pharmacists at community pharmacies do not have access to a shared list of a patient’s current medications, they may receive different CDSS alerts for the same patient [7]. In addition, one of the goals with the new national medication list in Sweden is that it should reduce the number of errors and discrepancies in the list, which would then result in fewer non-relevant alerts from the EES. 

### 4.3. DRPs and the Benefits of CDSS and Medication Reviews for Children

In other settings it has previously been shown that CDSS can decrease prescribing errors and wrong doses in children [27,28]. Little is known about the effects of using a CDSS for this population in community pharmacies. This study shows an increasing use of the system and adds knowledge about what kind of alerts are generated and resolved. Even though it is likely that the increasing use of the system would contribute to fewer actual DRPs among children, we cannot draw that conclusion. 

A previous study [32], which focused on clinically relevant potential interactions of class D and C, generated for the Swedish pediatric population (ages 0–17 years), showed that 0.14% had potential D interactions and 1.3% had potential C interactions. In the current study, DDIs represented 18% of all alerts for pediatric patients, where the most uncommon type of generated DDI alert was D interaction followed by C interaction.

Children are a distinct and heterogeneous group of patients that differ from adults regarding pharmacokinetics, pharmacodynamics, and medicine-related toxicity [20]. “High dose pediatric” being the most common type of alert (30.3% of all alerts) can therefore be explained by insufficient evidence-based treatment recommendations for many drugs for children [21]. Furthermore, no in-depth study has been performed to study the most common drugs that generated the warning alert “therapy duplication” or clinical significance.

The most common DRPs in prescribing among pediatric outpatients aged 0 to 16 years in Vietnam is “dose selection” and “dose timing relative to meals”, according to a study from 2021 [23]. In line with this study, “high dose pediatric” was the most generated alert. Children having a specific pharmacokinetic and varying weight, as well as being of different ages, indicates the importance of calculating the dose based on weight and age to individualize the treatment [23]. EES does not have access to the patients’ weight in the pharmacy dispensing system, instead estimations are made based on the age of the patient. 

### 4.4. Alerts Being Closed

The proportion of alerts being closed for the pediatric population is about 2.8% (week 11, 2022), which is quite similar to the findings for the total population in a previous study [7], 2.1% (week 11, 2019). Although this is a very low number, it is in line with some of the international research showing alert override rates as high as 98% in some studies [33]. Furthermore, the actual number of alerts being handled by pharmacists may be higher than the proportion of closed alerts shown in this study. According to a study from 2020, pharmacists often resolve matters related to a generated alert without closing it [7]. In the same study, pharmacists’ most common answer regarding why they have taken actions or handled an alert without closing it was that they wanted other pharmacists to see the same alert and lack of time, respectively [7]. Other reasons in the free text answers were that they forgot, taking too much time, technical issues, and uncertainty about the action, among others [7]. There is, however, no opportunity for a pharmacist to document the action taken to resolve an alert without closing the alert. The most common chosen reason for closing an alert was dialogue with patient for verification of the treatment. Similar results were shown in a previous study from 2020 [7].

Furthermore, the EES system is continuously updated, which might affect the number of alerts being generated and hence the proportion of alerts being resolved.

### 4.5. Method Discussion (Strengths and Weaknesses)

The type of obtained data determined the types of analyses that were possible. The aggregated data made it impossible to compare at the individual level related to age and gender. Nor was it possible to study the clinical relevance of alerts and the relationship with clinical outcomes due to the lack of health care data.

The amount of obtained data on the use of EES was however enough to study its change and extrapolate further use. Another strength of the study was the national perspective, including all pharmacies in Sweden. 

The statistics on pediatrics having prescriptions dispensed was of ages below 13 years, while the statistics on the number of EES alerts generated for pediatrics was of ages below 12 years. Thus, a completely accurate comparison could not be drawn. Furthermore, the proportion of alerts being closed were not possible to calculate for the total population, only for the pediatric population and only for one week due to the different types of obtained data for these groups.

“Supplementary review” was the second most common type of generated alert, which is a category that has been created to distinguish certain alerts within the other categories. Thus, measuring number of alerts in categories with a warning for a specific potential DRP produced uncertain results.

### 4.6. Future Research

The study period was decided to take place from week 11 of year 2020 to week 11 of year 2022. Thus, no focus has been placed on studying the potential effects of the pandemic (COVID-19), and with the first Swedish case being confirmed 31 January 2020, data after this date have only been analyzed. Future studies may fill in this knowledge gap. Furthermore, insufficient data has been obtained to study any effect of the latest focus week (week 9, 2021). Thus, there is room for further research that may provide valuable knowledge about the use of EES.

No analyses have been carried out on the pharmacists’ reasoning to the kinds of actions that were taken to resolve the generated alerts. Analyses of which the most common type of combined drugs that generated the signal “interactions” was not possible either. Future studies should investigate this, and the clinical relevance of the generated alerts. Pediatrics are a very vulnerable group, and so a great deal of focus should be put on understanding the potential DRPs that are generated using the CDSS EES.

## 5. Conclusions

The use of EES, i.e., the number of EES analyses, has increased for both the total population and the pediatric population. The most common type of generated alert for a potential DRP among children aged 0–12 years was high dose warning for pediatric patients. The results regarding which types of alert are the most common may contribute to an increased prescriber awareness of important potential DRPs. The most common type of alert for a potential DRP that was resolved was therapy duplication (45,8%). The two most common reasons for closing an alert are dialogue with patient for verification of the treatment and pharmaceutical assessment. The clinical effect from using this system is however unclear, and the clinical relevance of the generated alerts for pediatric patients cannot be assessed. The lack of patient-specific information contributes to the difficulty of drawing any conclusions. Despite the number of closed alerts also having increased, it is still very low.

## Figures and Tables

**Table 1:** Description of data and statistics, time, and population.

Data	Description	Time Period
Number of EES analyses	Number of times EES is used (calculated once/unique individual/pharmacy/day). *	Per week from week 11 of 2020 to week 11 of year 2022. (T&P)
Individuals having prescriptions dispensed	Number of individuals having prescriptions dispensed (calculated once/unique individual/pharmacy/day). *	Per week from week 11 of 2020 to week 11 of year 2022. (T&P)
Proportion of individuals getting an EES analysis	Number of EES analyses/Individuals having prescriptions dispensed (%). **	Per week (week 11 and 36 of 2020, week 11 and 36 of 2021, and week 11 of 2022). (T&P)
Number of EES alerts	Total number of alerts from EES. Each time a pharmacist presses the EES button, EES analyses the patient’s prescriptions and may generate a number of alerts. *	Week 11 of 2022 (P)
Average number of alerts per EES analysis	Number of EES alerts/number of EES analyses. The average number of generated alerts each time EES is used. **	Week 11 of 2022 (P)
Closed (resolved) EES alerts	Total number of closed alerts from EES. The pharmacist can close an alert after resolving it. *	Per week from week 11 of 2020 to week 11 of year 2022. (T&P)
Proportion of alerts being closed	Number of closed alerts/number of alerts (%). **	Week 11 of 2022 (P)
Type of alert generated, resolved, and documented action	The pharmacist can close an alert after resolving it and provide the reason for closing it according to the eHealth Agency’s available reasons. *	Week 11 of 2022 (P)

* Statistics from eHealth Agency, ** Calculation by researchers. (T&P) = Data for both the total population and the pediatric population (ages 0–12 years at the time). (P) = Data for only the Pediatric population (ages 0–12 years at the time).

**Table 2:** Number and proportion of EES analyses during the reference weeks of the total population (patients of all ages) and the pediatric population (ages 0–12 years). Number and proportion of alerts being resolved during week 11 of year 2022 of the pediatric population (ages 0–12 years).

		Week 11 2020	Week 36 2020	Week 11 2021	Week 36 2021	Week 11 2022
Total population	Number of EES analyses	290,678	345,003	466,984	531,675	576,510
	Individuals having prescriptions dispensed	1,227,797	953,236	1,004,752	980,988	1,019,349
	Proportion of individuals receiving an EES analysis (%)	24%	36%	46%	54%	57%
0–12 years old	Number of EES analyses	10,257	12,015	20,051	22,000	28,748
	Individuals having prescriptions dispensed *	69,042	37,417	41,337	41,475	50,827
	Proportion of individuals receiving an EES analysis (%)	15%	32%	49%	53%	57%
	Number of closed EES alerts	576	290	461	479	858

* For details and calculations, see Table 1.

**Table 3:** Generated alerts. Number and proportion of generated alerts and of alerts being closed within each category (week 11, 2022, pediatric ages 0–12 years).

Alert Categories	Number of Alerts	Proportion of All Alerts (%)	Number of Closed Alerts	Proportion Being Closed (%)
High dose pediatric	9339	30.3	238	2.55
Therapy duplication	7779	25.2	358	4.6
Age warning pediatric	7328	23.8	144	1.97
Drug–drug interactions	5605	18.2	27	0.48
Supplementary review	531	1.7	9	1.69
High dose	227	0.74	5	2.2
**Total**	30,809	100	781	2.5

## Data Availability

Restrictions apply to the availability of these data. Data were obtained from the Swedish eHealth Agency and can be made available (in aggregated form) from the authors with the permission of the Swedish eHealth Agency.

## Supplementary Materials

The following supporting information can be downloaded at: https://www.mdpi.com/article/10.3390/pharmacy11010035/s1, Figure S1: Comparison between the numbers of Electronic Expert Support (EES) analyses of the total population and the pediatric population, and Figure S2: Comparison between the numbers of alerts resolved of the total population and the pediatric population.
