# Supplementary material for: Potential Drug-Related Problems in Pediatric Patients—Describing the Use of a Clinical Decision Support System at Pharmacies in Sweden
Source: Pharmacy (Basel). 2023 Feb 14;11(1):35. doi: 10.3390/pharmacy11010035 (PMC9967379; doi:10.3390/pharmacy11010035)
Supplement: Supplementary file 1 [file pharmacy-11-00035-s001.zip › pharmacy-2043216-supplementary.pdf]

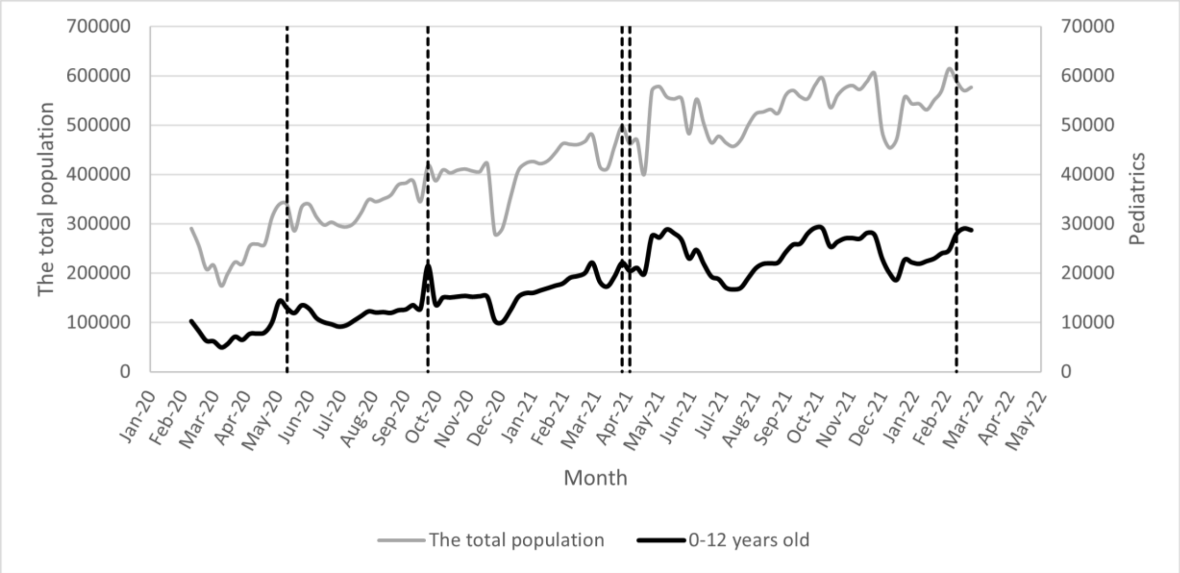

Figure S1. Comparison between the numbers of Electronic Expert Support (EES) analyses of the total population and the pediatric population ages 0–12 years per week between week 11, 2020 and week 53, 2020 (month = Mar-20–Dec-2020) ; week 1, 2021 and week 52, 2021 (month = Jan-21–Dec-21) ; week 1, 2022 and week 11, 2022 (month = Jan-22–Mar-22). Note, different values on the y-axes.

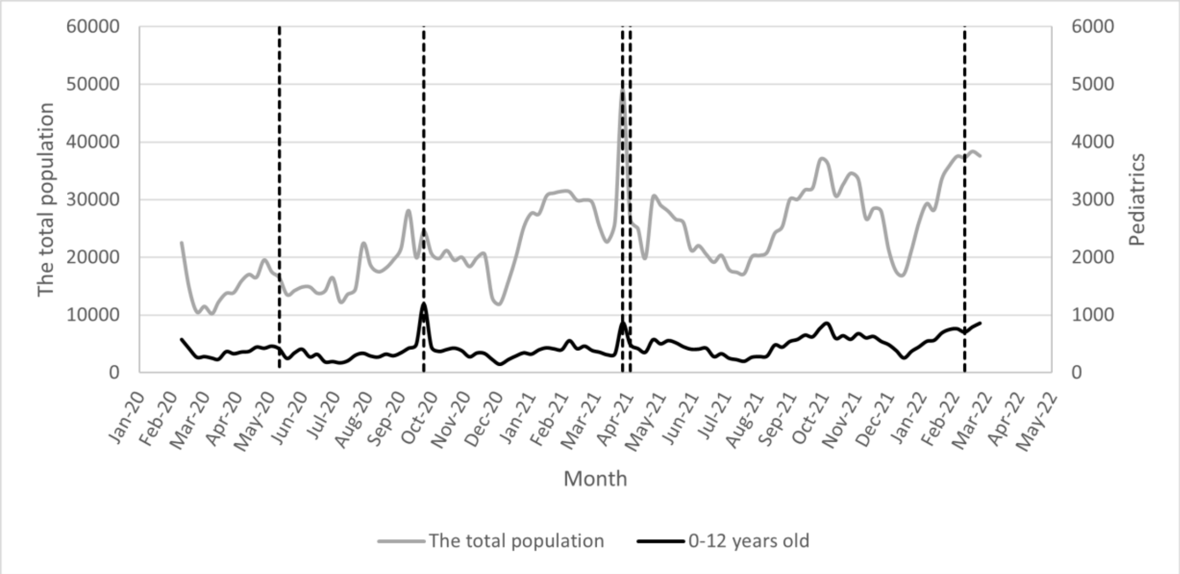

Figure S2. Comparison between the numbers of alerts resolved, i.e., alerts being closed, of the total population and the pediatric population ages 0–12 years per week between week 11, 2020 and week 53, 2020 (month = Mar-20–Dec-2020) ; week 1, 2021 and week 52, 2021 (month = Jan-21–Dec-21) ; week 1, 2022 and week 11, 2022 (month = Jan-22–Mar-22). Note, different values on the y-axes.
